# Supplementary material for: Genomic Characterization of the Titan-like Cell Producing Naganishia tulchinskyi, the First Novel Eukaryote Isolated from the International Space Station
Source: J Fungi (Basel). 2022 Feb 8;8(2):165. doi: 10.3390/jof8020165 (PMC8875396; doi:10.3390/jof8020165)
Supplement: Supplementary file 1 [file jof-08-00165-s001.zip › 6. jof-1548677-suppl updated.pdf]

Suppl Figures and Table legends

- **Supplementary Figure S1.** Maximum Likelihood phylogenetic tree, based on ITS regions, showing the phylogenetic relationship of *N. tulchinskyi* with closely related members of *Tremellomycetes*. Bootstrap values from 1,000 replications are shown at branch points. Bar, 0.02 substitution per site.
- **Supplementary Figure S2.** Maximum Likelihood phylogenetic tree, based on LSU sequences, showing the phylogenetic relationship of *N. tulchinskyi* with closely related members of *Tremellomycetes*. Bootstrap values from 1,000 replications are shown at branch points. Bar, 0.02 substitution per site.
- **Supplementary Figure S3.** Maximum Likelihood phylogenetic tree, based on SSU sequences, showing the phylogenetic relationship of *N. tulchinskyi* with closely related members of *Tremellomycetes*. Bootstrap values from 1,000 replications are shown at branch points. Bar, 0.01 substitution per site.
- **Supplementary Figure S4.** Maximum Likelihood phylogenetic tree, based on *cytB* sequences, showing the phylogenetic relationship of *N. tulchinskyi* with closely related members of *Tremellomycetes*. Bootstrap values from 1,000 replications are shown at branch points. Bar, 0.05 substitution per site.
- **Supplementary Figure S5.** Maximum Likelihood phylogenetic tree, based on *rpb*1 sequences, showing the phylogenetic relationship of *N. tulchinskyi* with closely related members of *Tremellomycetes*. Bootstrap values from 1,000 replications are shown at branch points. Bar, 0.10 substitution per site.
- **Supplementary Figure S6.** Maximum Likelihood phylogenetic tree, based on *rpb*2 sequences, showing the phylogenetic relationship of *N. tulchinskyi* with closely related members of *Tremellomycetes*. Bootstrap values from 1,000 replications are shown at branch points. Bar, 0.10 substitution per site.
- **Supplementary Figure S7.** Maximum Likelihood phylogenetic tree, based on *tef*1 sequences, showing the phylogenetic relationship of *N. tulchinskyi* with closely related members of *Tremellomycetes*. Bootstrap values from 1,000 replications are shown at branch points. Bar, 0.02 substitution per site.
- **Supplementary Figure S8.** SEM (A-C) and TEM (D-G) micrographs highlighting the unusual µG grown Titan-like cell morphology. Titan-like cells show large concave and convex budding scars (A-C). A daughter cell can be seen budding from the mother yeast cell (black arrow), and appears to be altering the shape of the mother cell's cell wall (B). Budding scars are shown to be peeling off of the yeast surfaces (white arrows) (A-C). This phenomenon is shown in TEM images, designated by white arrows (D-G). SEM scale bars are 10µm. TEM scale bars are 5µm (D), 1µm (E-F), and 0.5µm (G). The letter "N" distinguishes the nucleus (B,C, and E).
- **Supplementary Table S1.** Reducing all protein-coding gene products from the predicted proteome of the novel type strain IF6SW-B1^{T} to their most distinct Gene Ontology (GO) terms for Biological Process, Cellular Component, and Molecular Function. Frequency indicates the number of genes in UniProt associated with this term. Uniqueness is a measurement of average similarity of a term to all other GO terms, while dispensability measures the semantic distance between a term and all other GO terms.

Figure S1

ITS_ML tree

Mycobank species
Naganishia adeliensis
Naganishia albida
Naganishia albidosimilis
Naganishia antarctica
Naganishia bhutanensis
Naganishia brisbanensis
Naganishia cerealis
Naganishia diffuens
Naganishia floricola
Naganishia friedmannii
Naganishia globosa
Naganishia indica (no ITS)
Naganishia liquefaciens
Naganishia nivalis
Naganishia onofrii
Naganishia qatarensis
Naganishia randhawae
Naganishia uzbekistanensis
Naganishia vaughanmartiniae
Naganishia vishniacii

CBS species
Naganishia adeliensis
Naganishia albidosimilis
Naganishia antarctica
Naganishia bhutanensis
Naganishia diffuens
Naganishia friedmannii
Naganishia globosa
Naganishia randhawae
Naganishia uzbekistanensis
Naganishia vaughanmartiniae
Naganishia vishniacii

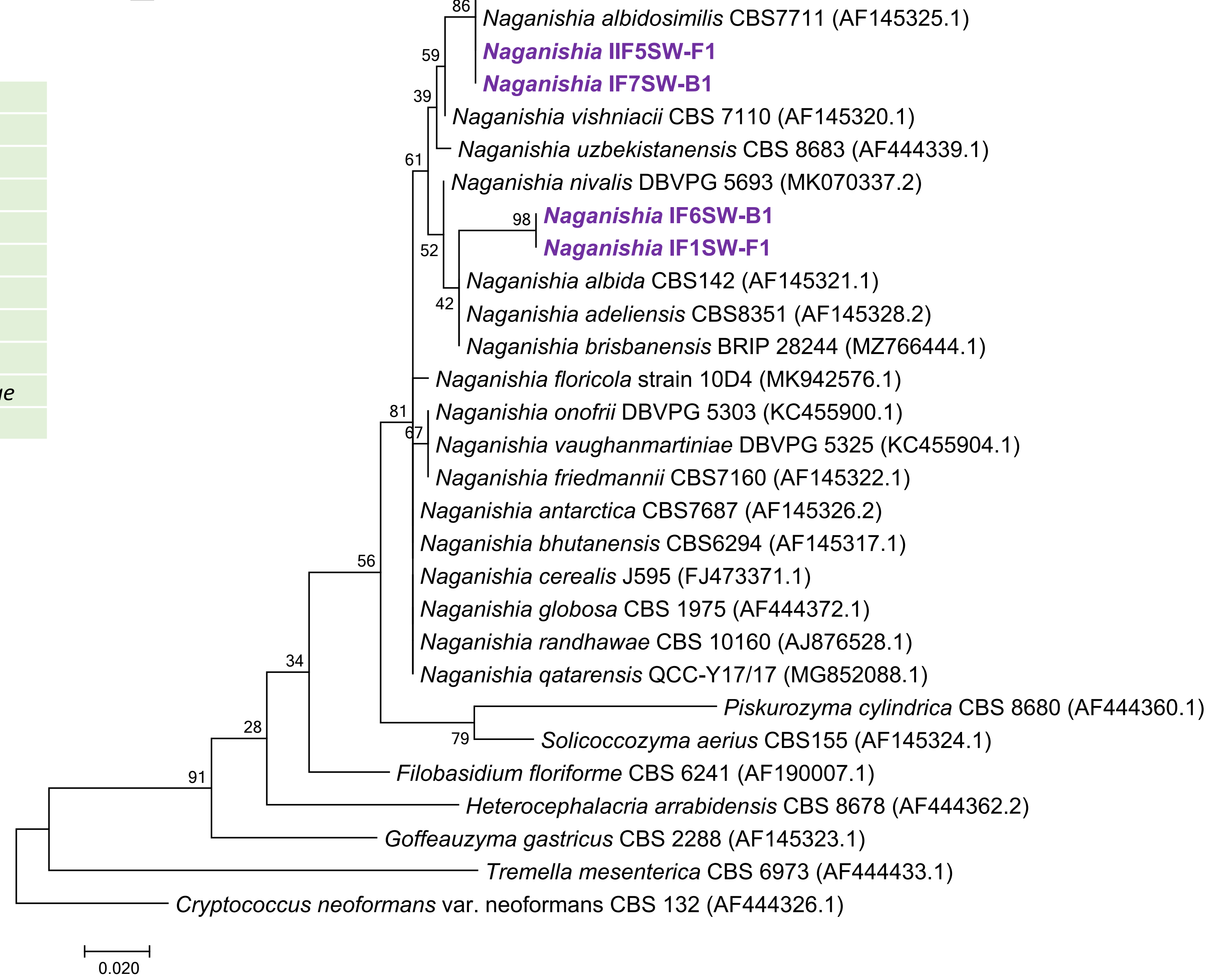

Figure S2

LSU_ML tree

Mycobank species

O	*Naganishia adeliensis*
O	*Naganishia albida*
O	*Naganishia albidosimilis*
O	*Naganishia antarctica*
O	*Naganishia bhutanensis*
O	*Naganishia cerealis*
O	*Naganishia diffuens*
O	*Naganishia friedmannii*
O	*Naganishia globosa*
X	*Naganishia indica*
O	*Naganishia liquefaciens*
X	*Naganishia nivalis*
O	*Naganishia onofrii*
X	*Naganishia qatarensis*
O	*Naganishia randhawae*
O	*Naganishia uzbekistanensis*
O	*Naganishia vaughanmartiniae*
O	*Naganishia vishniacii*

CBS species

- *Naganishia adeliensis*
- *Naganishia albidosimilis*
- *Naganishia antarctica*
- *Naganishia bhutanensis*
- *Naganishia diffuens*
- *Naganishia friedmannii*
- *Naganishia globosa*
- *Naganishia randhawae*
- *Naganishia uzbekistanensis*
- *Naganishia vaughanmartiniae*
- *Naganishia vishniacii*

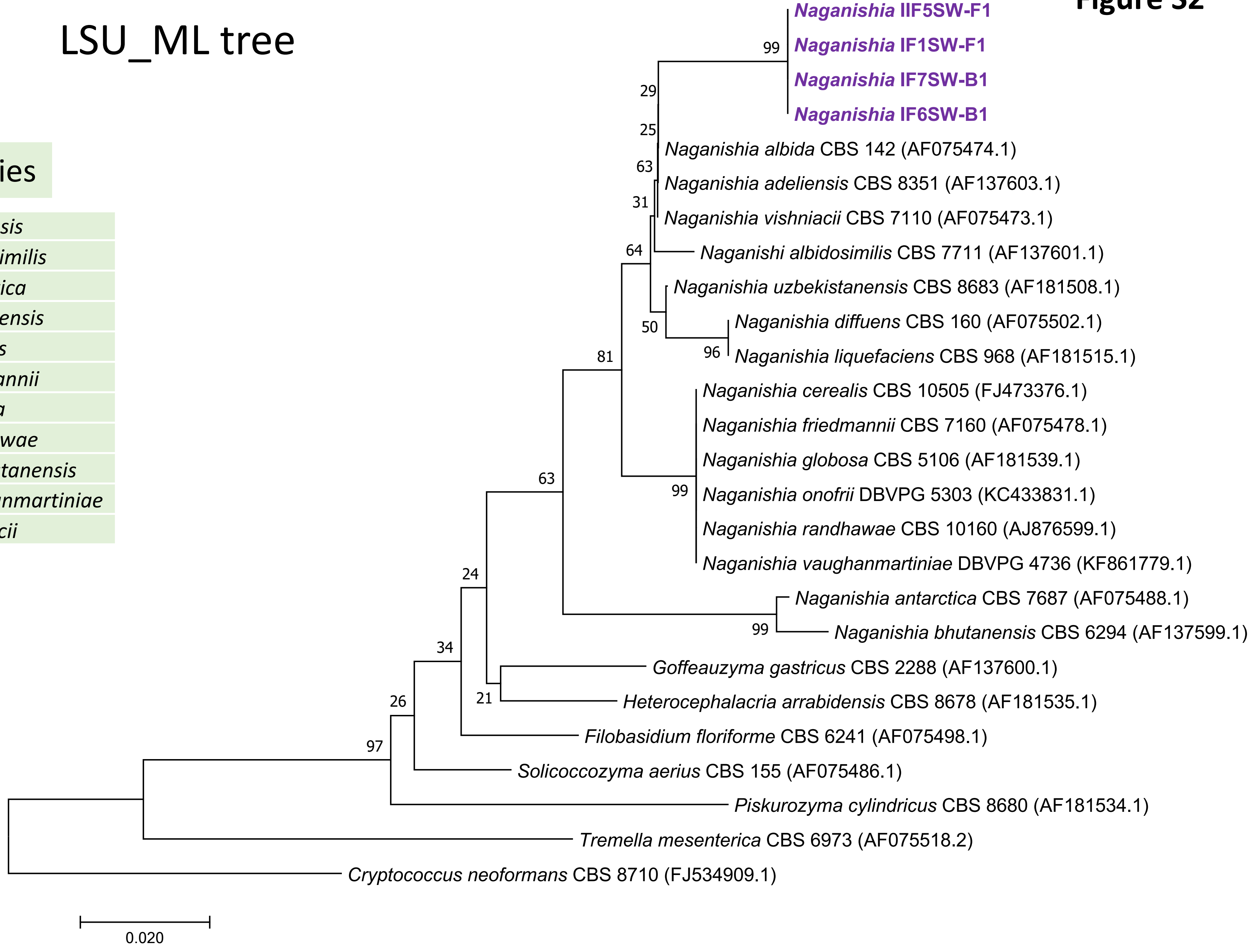

Figure S3

SSU_ML tree

	Mycobank species
O	*Naganishia adeliensis*
O	*Naganishia albida*
O	*Naganishia albidosimilis*
O	*Naganishia antarctica*
O	*Naganishia bhutanensis*
O	*Naganishia cerealis*
O	*Naganishia diffuens*
O	*Naganishia friedmannii*
O	*Naganishia globosa*
X	*Naganishia indica*
O	*Naganishia liquefaciens*
X	*Naganishia nivalis*
X	*Naganishia onofrii*
X	*Naganishia qatarensis*
X	*Naganishia randhawae*
O	*Naganishia uzbekistanensis*
X	*Naganishia vaughanmartiniae*
O	*Naganishia vishniacii*

CBS species
Naganishia adeliensis
Naganishia albidosimilis
Naganishia antarctica
Naganishia bhutanensis
Naganishia diffuens
Naganishia friedmannii
Naganishia globosa
Naganishia randhawae
Naganishia uzbekistanensis
Naganishia vaughanmartiniae
Naganishia vishniacii

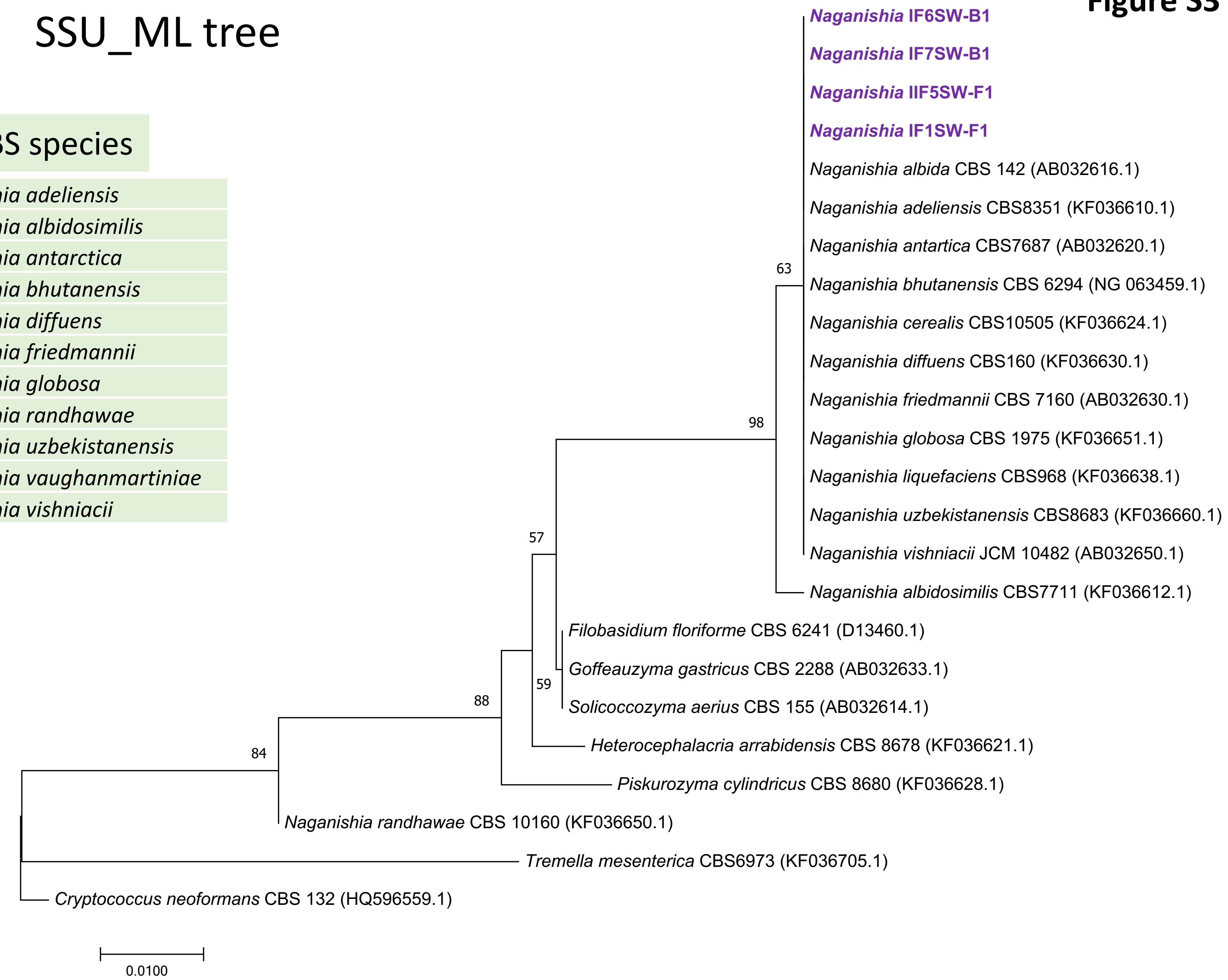

Figure S4

*CYTB*_ML tree

	Mycobank species
O	*Naganishia adeliensis*
O	*Naganishia albida*
O	*Naganishia albidosimilis*
O	*Naganishia antarctica*
O	*Naganishia bhutanensis*
O	*Naganishia cerealis*
O	*Naganishia diffuens*
O	*Naganishia friedmannii*
O	*Naganishia globosa*
X	*Naganishia indica*
O	*Naganishia liquefaciens*
X	*Naganishia nivalis*
X	*Naganishia onofrii*
X	*Naganishia qatarensis*
X	*Naganishia randhawae*
O	*Naganishia uzbekistanensis*
X	*Naganishia vaughanmartiniae*
O	*Naganishia vishniacii*

CBS species
Naganishia adeliensis
Naganishia albidosimilis
Naganishia antarctica
Naganishia bhutanensis
Naganishia diffuens
Naganishia friedmannii
Naganishia globosa
Naganishia randhawae
Naganishia uzbekistanensis
Naganishia vaughanmartiniae
Naganishia vishniacii

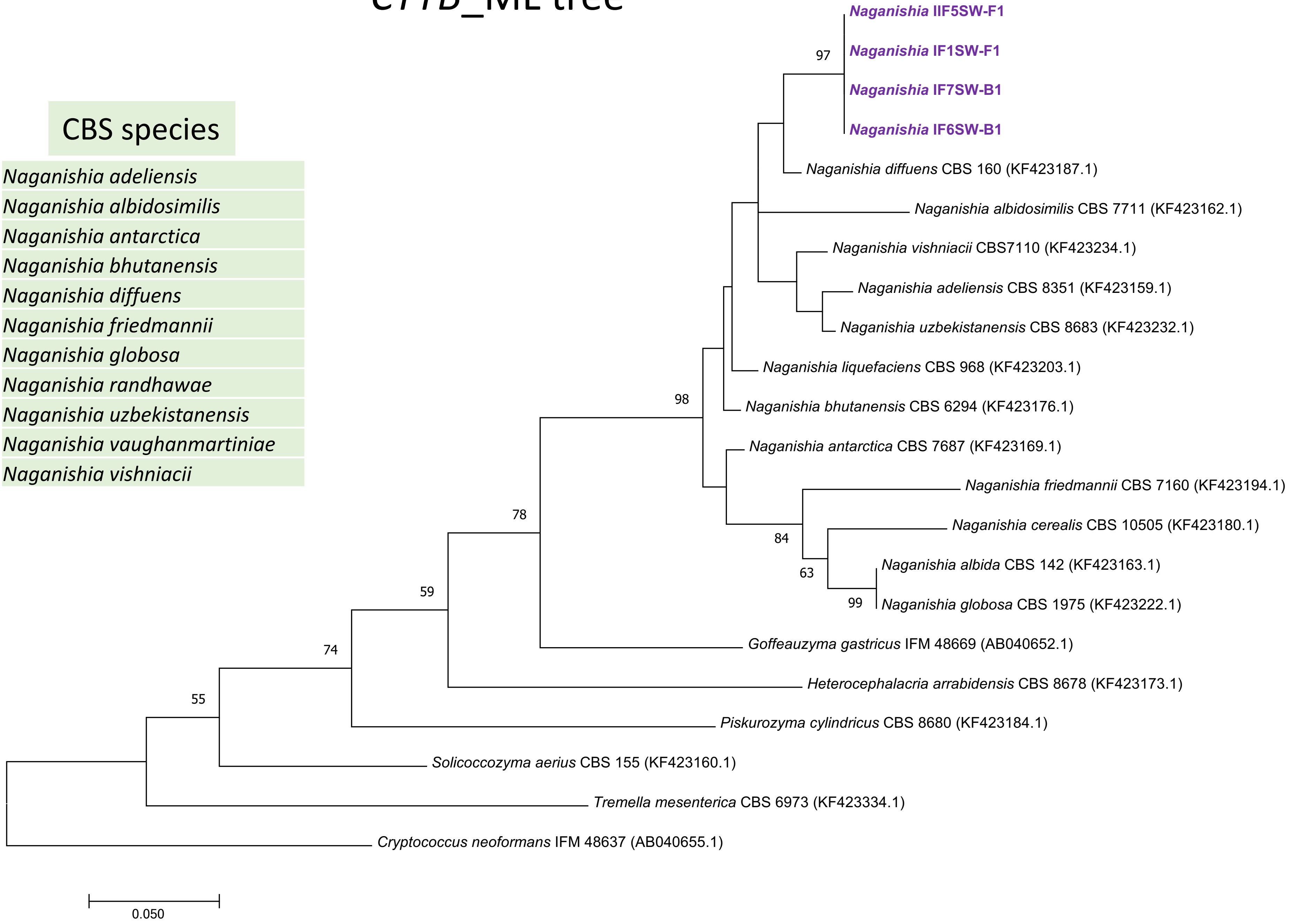

Figure S5

*RPB1*_ML tree

	Mycobank species	CBS species
O	*Naganishia adeliensis*	*Naganishia adeliensis*
O	*Naganishia albida*	*Naganishia albidosimilis*
O	*Naganishia albidosimilis*	*Naganishia antarctica*
O	*Naganishia antarctica*	*Naganishia bhutanensis*
O	*Naganishia bhutanensis*	*Naganishia diffuens*
O	*Naganishia cerealis*	*Naganishia friedmannii*
O	*Naganishia diffuens*	*Naganishia globosa*
O	*Naganishia friedmannii*	*Naganishia randhawae*
O	*Naganishia globosa*	*Naganishia uzbekistanensis*
X	*Naganishia indica*	*Naganishia vaughanmartiniae*
O	*Naganishia liquefaciens*	*Naganishia vishniacii*
X	*Naganishia nivalis*	
X	*Naganishia onofrii*	
X	*Naganishia qatarensis*	
X	*Naganishia randhawae*	
O	*Naganishia uzbekistanensis*	
X	*Naganishia vaughanmartiniae*	
O	*Naganishia vishniacii*	

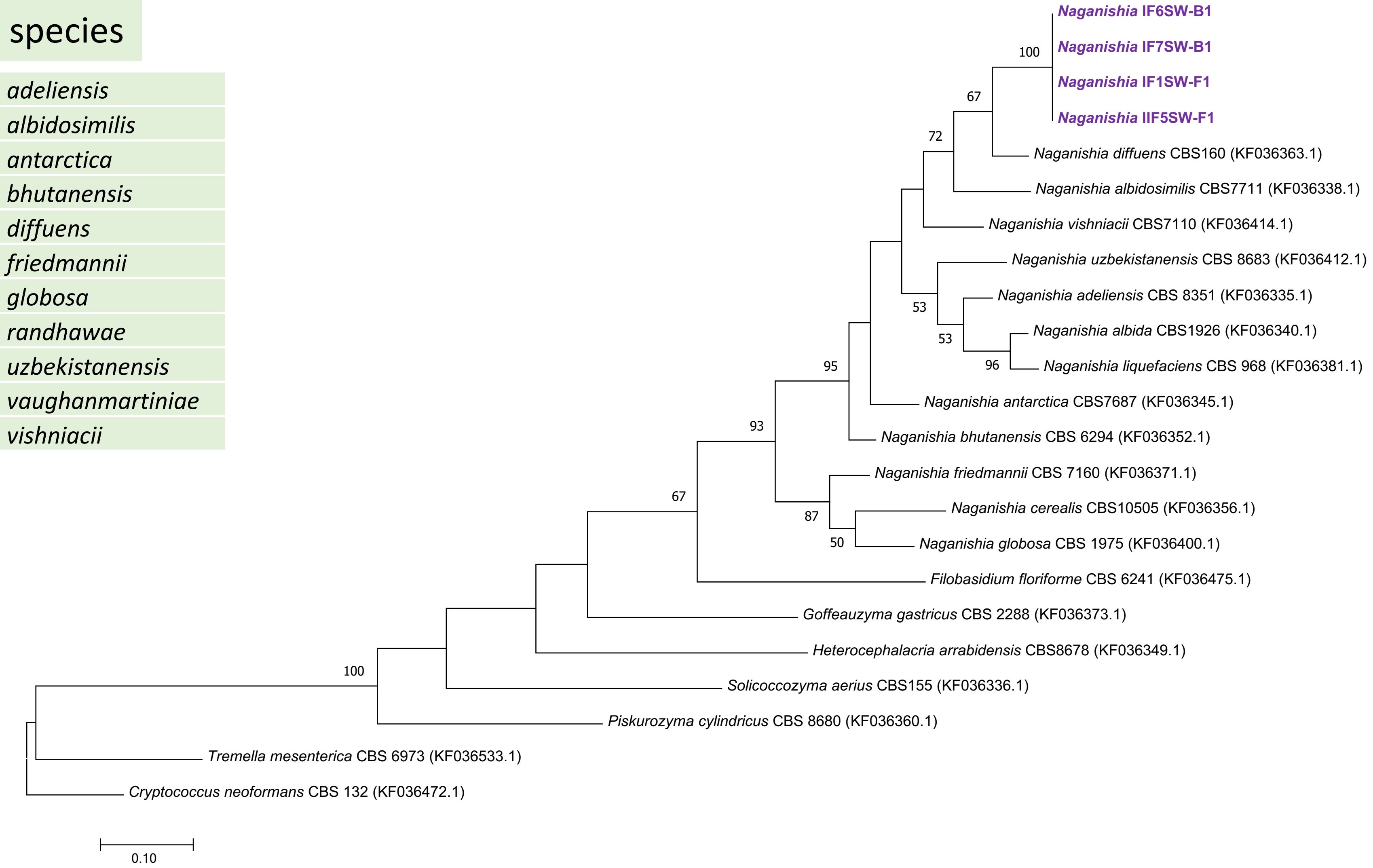

*RPB2*_ML tree

Figure S6

	Mycobank species
O	*Naganishia adeliensis*
O	*Naganishia albida*
O	*Naganishia albidosimilis*
O	*Naganishia antarctica*
O	*Naganishia bhutanensis*
X	*Naganishia cerealis*
O	*Naganishia diffuens*
O	*Naganishia friedmannii*
O	*Naganishia globosa*
X	*Naganishia indica*
O	*Naganishia liquefaciens*
X	*Naganishia nivalis*
X	*Naganishia onofrii*
x	*Naganishia qatarensis*
x	*Naganishia randhawae*
O	*Naganishia uzbekistanensis*
x	*Naganishia vaughanmartiniae*
x	*Naganishia vishniacii*

CBS species
Naganishia adeliensis
Naganishia albidosimilis
Naganishia antarctica
Naganishia bhutanensis
Naganishia diffuens
Naganishia friedmannii
Naganishia globosa
Naganishia randhawae
Naganishia uzbekistanensis
Naganishia vaughanmartiniae
Naganishia vishniacii

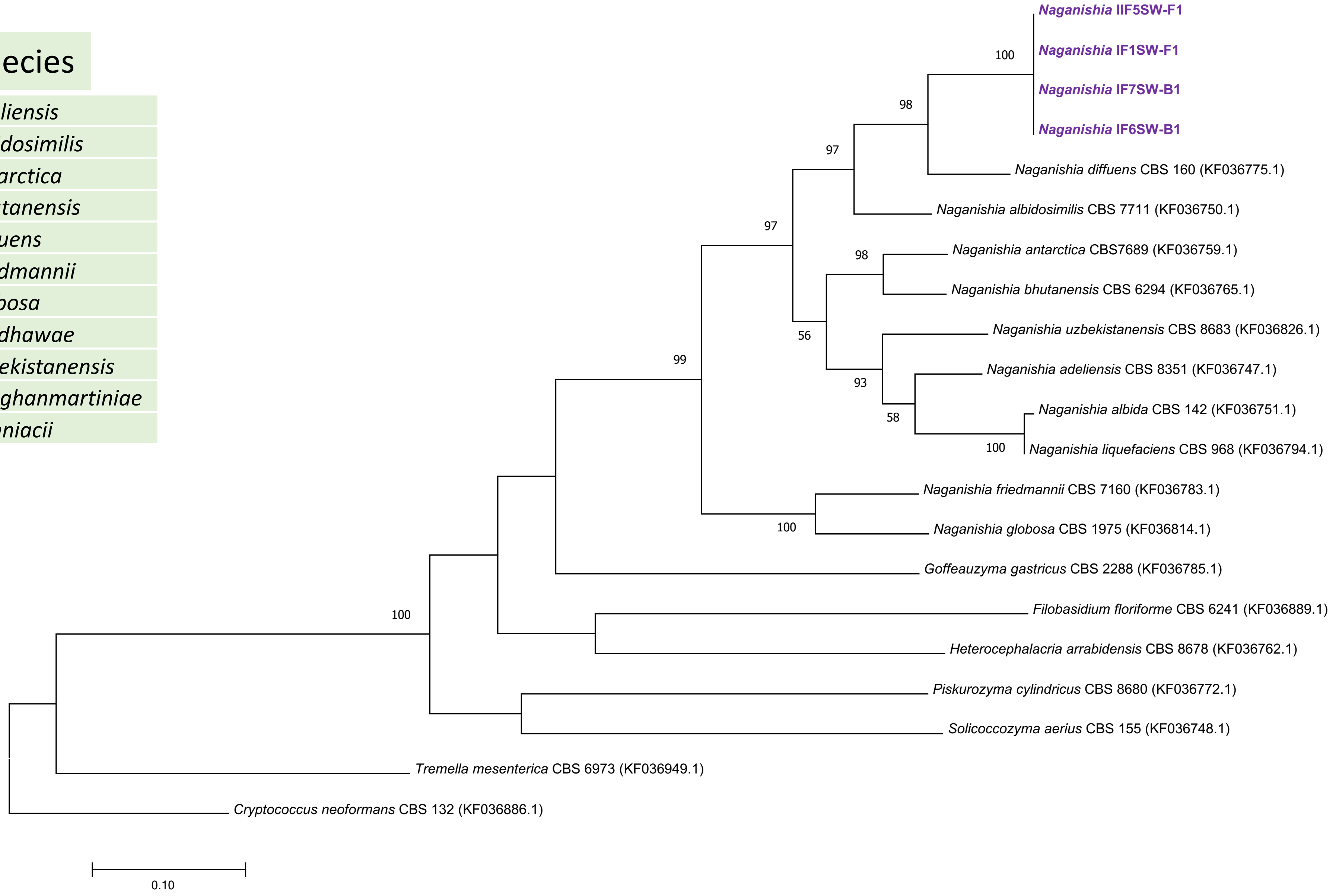

*TEF1*_ML tree

Figure S7

	Mycobank species
O	*Naganishia adeliensis*
O	*Naganishia albida*
O	*Naganishia albidosimilis*
O	*Naganishia antarctica*
O	*Naganishia bhutanensis*
O	*Naganishia cerealis*
O	*Naganishia diffuens*
O	*Naganishia friedmannii*
O	*Naganishia globosa*
X	*Naganishia indica*
O	*Naganishia liquefaciens*
X	*Naganishia nivalis*
X	*Naganishia onofrii*
x	*Naganishia qatarensis*
x	*Naganishia randhawae*
O	*Naganishia uzbekistanensis*
x	*Naganishia vaughanmartiniae*
x	*Naganishia vishniacii*

CBS species
Naganishia adeliensis
Naganishia albidosimilis
Naganishia antarctica
Naganishia bhutanensis
Naganishia diffuens
Naganishia friedmannii
Naganishia globosa
Naganishia randhawae
Naganishia uzbekistanensis
Naganishia vaughanmartiniae
Naganishia vishniacii

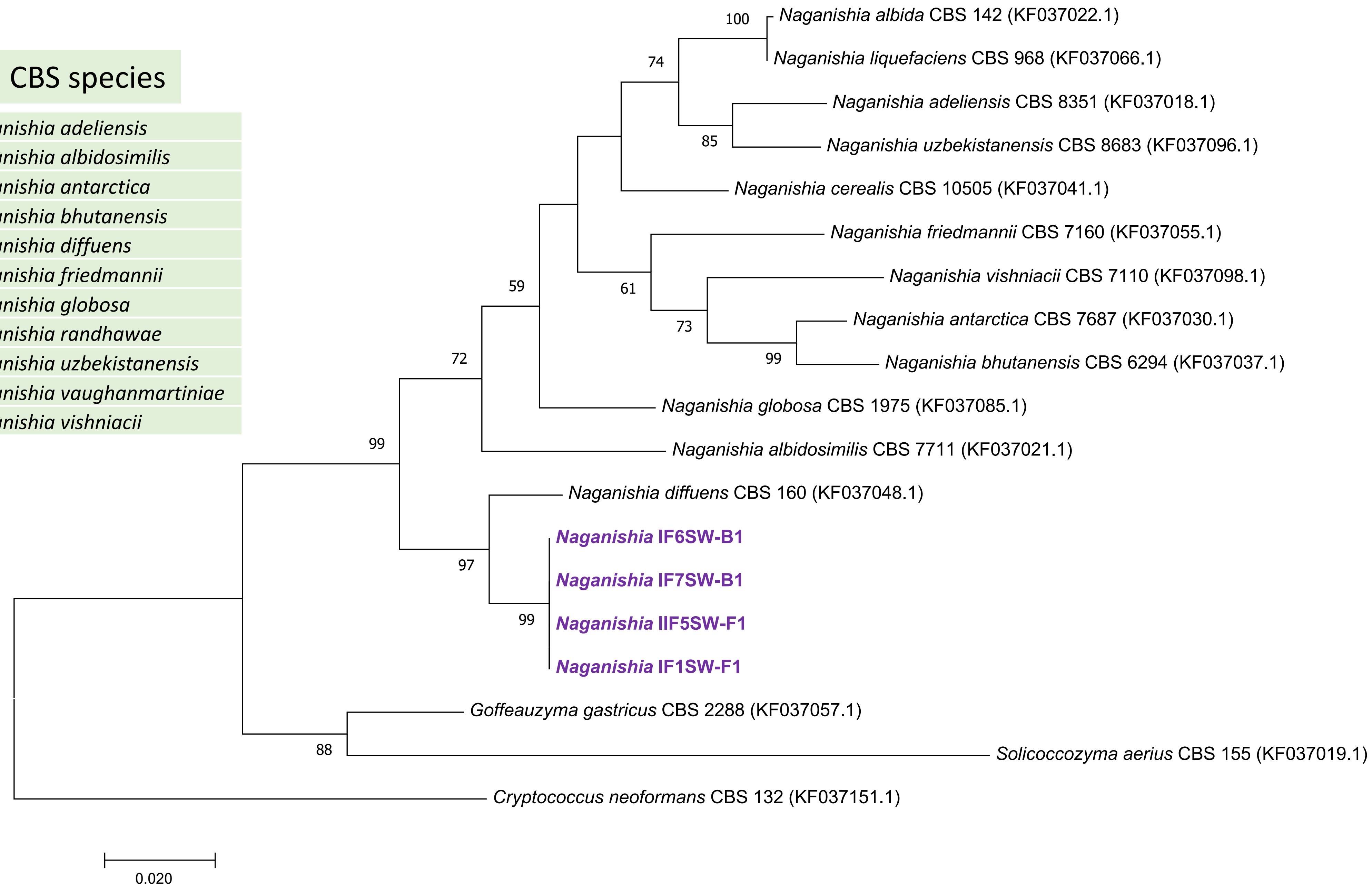

Figure S8

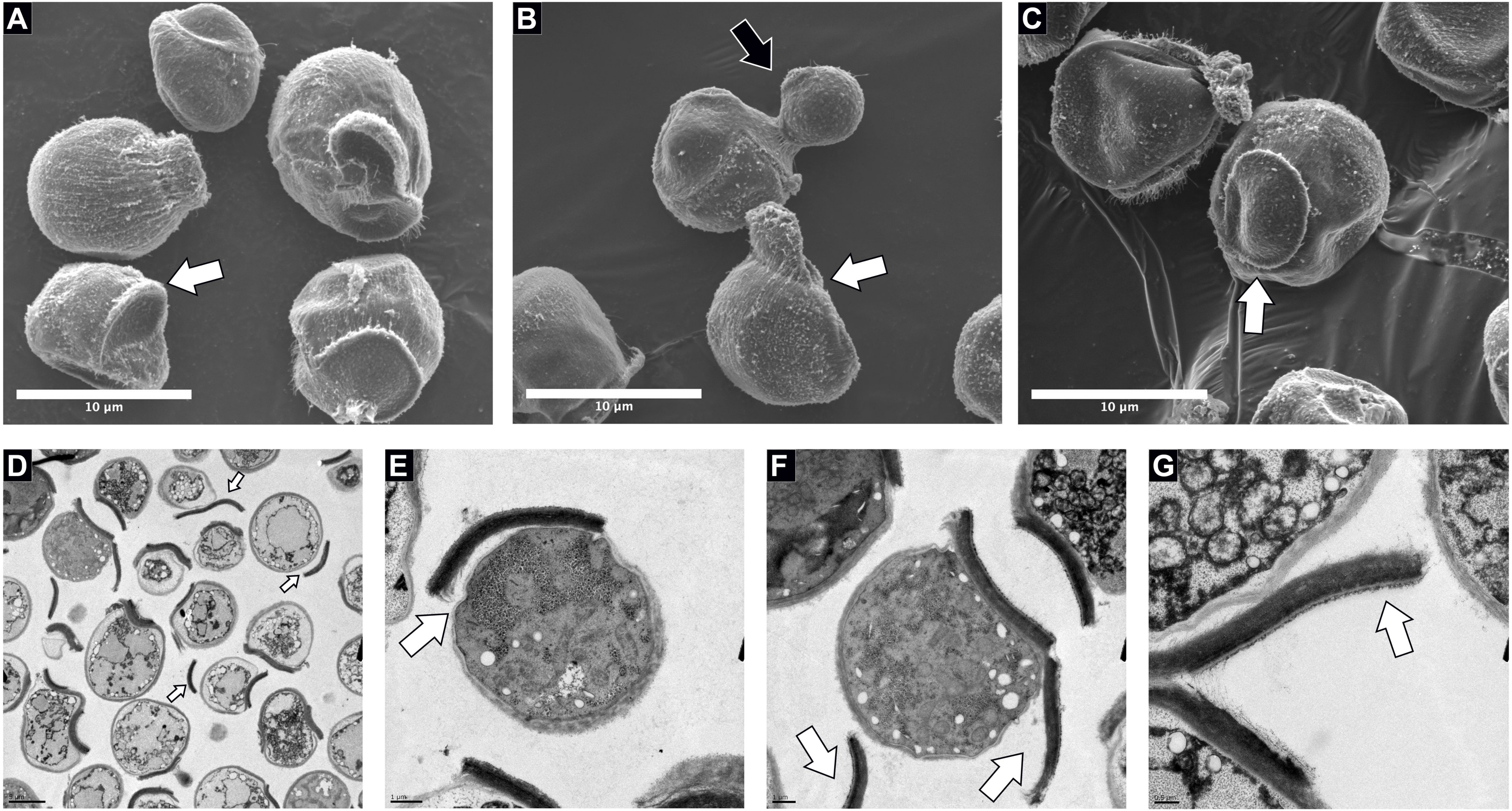

Term ID	Name	Frequency	Uniqueness	Dispensability
GO:0000902	cell morphogenesis	0.37%	1	0
GO:0007017	microtubule-based process	1.61%	0.99	0
GO:0009411	response to UV	0.15%	0.96	0
GO:0046034	ATP metabolic process	1.58%	0.94	0
GO:2001256	regulation of store-operated calcium entry	0.03%	0.97	0
GO:0031144	proteasome localization	0.03%	0.95	0
GO:0007018	microtubule-based movement	0.38%	0.98	0.01
GO:0006458	'de novo' protein folding	0.43%	0.99	0.01
GO:0007031	peroxisome organization	0.86%	0.93	0.01
GO:0061640	cytoskeleton-dependent cytokinesis	1.19%	0.97	0.01
GO:0006457	protein folding	1.97%	0.99	0.01
GO:0006662	glycerol ether metabolic process	0.03%	0.85	0.04
GO:0006072	glycerol-3-phosphate metabolic process	0.07%	0.87	0.04
GO:0000103	sulfate assimilation	0.15%	0.91	0.04
GO:0006081	cellular aldehyde metabolic process	0.60%	0.93	0.05
GO:0006099	tricarboxylic acid cycle	0.50%	0.92	0.05
GO:0009308	amine metabolic process	0.43%	0.88	0.05
GO:0005991	trehalose metabolic process	0.18%	0.9	0.06
GO:0032259	methylation	1.84%	0.94	0.06
GO:0006914	autophagy	2.90%	0.89	0.06
GO:0005975	carbohydrate metabolic process	3.86%	0.91	0.08
GO:0032780	negative regulation of ATPase activity	0.05%	0.96	0.09
GO:0009058	biosynthetic process	25.36%	0.91	0.1
GO:0055088	lipid homeostasis	0.56%	0.95	0.11
GO:0006629	lipid metabolic process	5.30%	0.9	0.11
GO:0018298	protein-chromophore linkage	0.02%	0.86	0.11
GO:0045329	carnitine biosynthetic process	0.02%	0.82	0.11
GO:0016480	negative regulation of transcription by RNA polymerase III	0.08%	0.95	0.12
GO:0015706	nitrate transport	0.02%	0.94	0.12
GO:0042558	pteridine-containing compound metabolic process	0.30%	0.83	0.14
GO:0009272	fungal-type cell wall biogenesis	1.41%	0.93	0.14
GO:0071586	CAAX-box protein processing	0.03%	0.84	0.15
GO:0006260	DNA replication	2.07%	0.79	0.17
GO:0009966	regulation of signal transduction	2.32%	0.94	0.17
GO:0043631	RNA polyadenylation	0.43%	0.8	0.18
GO:0016559	peroxisome fission	0.15%	0.94	0.2
GO:0061817	endoplasmic reticulum-plasma membrane tethering	0.08%	0.9	0.2
GO:0042407	cristae formation	0.17%	0.93	0.2
GO:0006596	polyamine biosynthetic process	0.07%	0.79	0.21
GO:0019915	lipid storage	0.12%	0.94	0.21
GO:0034198	cellular response to amino acid starvation	0.28%	0.94	0.21
GO:1901642	nucleoside transmembrane transport	0.07%	0.94	0.21
GO:0030261	chromosome condensation	0.33%	0.92	0.22
GO:0007034	vacuolar transport	3.03%	0.93	0.22
GO:0006480	N-terminal protein amino acid methylation	0.03%	0.85	0.22
GO:0006807	nitrogen compound metabolic process	47.24%	0.9	0.22
GO:0042176	regulation of protein catabolic process	0.43%	0.95	0.23
GO:0019538	protein metabolic process	23.24%	0.79	0.23
GO:0048268	clathrin coat assembly	0.07%	0.93	0.23
GO:0006101	citrate metabolic process	0.03%	0.83	0.24
GO:0042256	mature ribosome assembly	0.10%	0.92	0.24
GO:0006779	porphyrin-containing compound biosynthetic process	0.28%	0.75	0.25
GO:0018342	protein prenylation	0.13%	0.83	0.25
GO:0006370	7-methylguanosine mRNA capping	0.13%	0.8	0.26
GO:0051103	DNA ligation involved in DNA repair	0.02%	0.82	0.26
GO:0006744	ubiquinone biosynthetic process	0.23%	0.78	0.27
GO:0006891	intra-Golgi vesicle-mediated transport	0.53%	0.92	0.27
GO:0001522	pseudouridine synthesis	0.27%	0.78	0.27
GO:0006275	regulation of DNA replication	0.90%	0.95	0.28
GO:0007010	cytoskeleton organization	3.86%	0.92	0.28
GO:0007015	actin filament organization	1.04%	0.92	0.29
GO:0000413	protein peptidyl-prolyl isomerization	0.27%	0.82	0.29
GO:0000959	mitochondrial RNA metabolic process	0.75%	0.79	0.3
GO:0042128	nitrate assimilation	0.05%	0.81	0.32
GO:0006606	protein import into nucleus	1.01%	0.91	0.32
GO:1990426	mitotic recombination-dependent replication fork processing	0.02%	0.8	0.33
GO:0097193	intrinsic apoptotic signaling pathway	0.02%	0.93	0.33
GO:0051259	protein complex oligomerization	0.13%	0.93	0.33
GO:0070072	vacuolar proton-transporting V-type ATPase complex assembly	0.08%	0.93	0.33
GO:0046417	chorismate metabolic process	0.08%	0.81	0.34
GO:0006414	translational elongation	0.56%	0.74	0.34
GO:0015074	DNA integration	0.83%	0.78	0.35
GO:0032784	regulation of DNA-templated transcription, elongation	0.91%	0.94	0.35
GO:0044237	cellular metabolic process	54.67%	0.89	0.35
GO:0009451	RNA modification	1.84%	0.75	0.35
GO:0016311	dephosphorylation	2.07%	0.86	0.36
GO:0007005	mitochondrion organization	4.51%	0.92	0.36
GO:0006139	nucleobase-containing compound metabolic process	24.52%	0.75	0.37
GO:0016579	protein deubiquitination	0.53%	0.8	0.37
GO:0006419	alanyl-tRNA aminoacylation	0.02%	0.7	0.38
GO:0006011	UDP-glucose metabolic process	0.08%	0.74	0.38
GO:0006508	proteolysis	7.00%	0.8	0.38
GO:0034599	cellular response to oxidative stress	1.84%	0.93	0.39
GO:0016192	vesicle-mediated transport	7.09%	0.92	0.39
GO:0043248	proteasome assembly	0.53%	0.92	0.39
GO:0045901	positive regulation of translational elongation	0.07%	0.94	0.39

Term ID	Name	Frequency	Uniqueness	Dispensability
GO:0005576	extracellular region	1.96%	1	0
GO:0005777	peroxisome	1.51%	0.98	0
GO:0019867	outer membrane	1.92%	0.93	0
GO:0005618	cell wall	2.27%	0.97	0
GO:0016020	membrane	34.69%	1	0
GO:0005737	cytoplasm	73.82%	0.99	0.01
GO:0005853	eukaryotic translation elongation factor 1 complex	0.02%	0.87	0.01
GO:0005840	ribosome	4.16%	0.88	0.01
GO:0005801	cis-Golgi network	0.28%	0.94	0.02
GO:0005759	mitochondrial matrix	3.98%	0.87	0.02
GO:0005829	cytosol	16.60%	0.98	0.03
GO:0005634	nucleus	39.66%	0.97	0.04
GO:0005739	mitochondrion	19.85%	0.97	0.07
GO:0031251	PAN complex	0.03%	0.86	0.08
GO:0005835	fatty acid synthase complex	0.03%	0.86	0.08
GO:0042555	MCM complex	0.10%	0.85	0.09
GO:0032299	ribonuclease H2 complex	0.05%	0.78	0.09
GO:0016272	prefoldin complex	0.10%	0.85	0.1
GO:0005786	signal recognition particle, endoplasmic reticulum targeting	0.10%	0.81	0.1
GO:0005852	eukaryotic translation initiation factor 3 complex	0.12%	0.85	0.1
GO:0008180	COP9 signalosome	0.12%	0.79	0.1
GO:0045239	tricarboxylic acid cycle enzyme complex	0.17%	0.85	0.1
GO:0030117	membrane coat	0.58%	0.7	0.15
GO:0009316	3-isopropylmalate dehydratase complex	0.02%	0.8	0.18
GO:0009349	riboflavin synthase complex	0.02%	0.79	0.21
GO:0016021	integral component of membrane	20.92%	0.91	0.25
GO:0072686	mitotic spindle	0.32%	0.87	0.25
GO:0000178	exosome (RNase complex)	0.27%	0.76	0.25
GO:0005960	glycine cleavage complex	0.05%	0.76	0.27
GO:0000214	tRNA-intron endonuclease complex	0.07%	0.74	0.28
GO:0005760	gamma DNA polymerase complex	0.02%	0.69	0.29
GO:0000776	kinetochore	1.19%	0.82	0.3
GO:0005849	mRNA cleavage factor complex	0.33%	0.77	0.3
GO:0030289	protein phosphatase 4 complex	0.05%	0.74	0.31
GO:0031417	NatC complex	0.05%	0.74	0.31
GO:0048500	signal recognition particle	0.10%	0.81	0.31
GO:0005956	protein kinase CK2 complex	0.07%	0.78	0.32
GO:0033588	elongator holoenzyme complex	0.10%	0.77	0.33
GO:0000145	exocyst	0.15%	0.81	0.33
GO:0016592	mediator complex	0.46%	0.77	0.34
GO:0005783	endoplasmic reticulum	11.42%	0.93	0.34
GO:0030015	CCR4-NOT core complex	0.17%	0.76	0.35
GO:0005741	mitochondrial outer membrane	1.77%	0.82	0.36
GO:0005732	sno(s)RNA-containing ribonucleoprotein complex	0.55%	0.78	0.36
GO:0005694	chromosome	8.15%	0.87	0.39
GO:0005663	DNA replication factor C complex	0.08%	0.78	0.4

Term ID	Name	Frequency	Uniqueness	Dispensability
GO:0003774	cytoskeletal motor activity	0.27%	1	0
GO:0003777	microtubule motor activity	0.17%	0.98	0
GO:0005049	nuclear export signal receptor activity	0.05%	0.97	0
GO:0030246	carbohydrate binding	0.56%	0.99	0
GO:0050385	ureidoglycolate lyase activity	0.02%	0.86	0
GO:0030955	potassium ion binding	0.03%	0.97	0.03
GO:0032051	clathrin light chain binding	0.03%	0.99	0.03
GO:0030942	endoplasmic reticulum signal peptide binding	0.05%	0.98	0.03
GO:0008061	chitin binding	0.08%	0.98	0.03
GO:0071933	Arp2/3 complex binding	0.08%	0.98	0.03
GO:0005536	glucose binding	0.12%	0.97	0.03
GO:0020037	heme binding	0.43%	0.97	0.03
GO:0043015	gamma-tubulin binding	0.03%	0.98	0.07
GO:0080132	fatty acid alpha-hydroxylase activity	0.02%	0.83	0.07
GO:0017025	TBP-class protein binding	0.30%	0.98	0.08
GO:0019903	protein phosphatase binding	0.27%	0.98	0.09
GO:0030276	clathrin binding	0.35%	0.98	0.09
GO:0004334	fumarylacetoacetase activity	0.02%	0.85	0.09
GO:0004452	isopentenyl-diphosphate delta-isomerase activity	0.02%	0.86	0.09
GO:0004637	phosphoribosylamine-glycine ligase activity	0.02%	0.84	0.09
GO:0008410	CoA-transferase activity	0.02%	0.84	0.09
GO:0051087	chaperone binding	0.43%	0.98	0.1
GO:0046982	protein heterodimerization activity	0.56%	0.98	0.1
GO:1990610	acetolactate synthase regulator activity	0.02%	0.98	0.11
GO:0000340	RNA 7-methylguanosine cap binding	0.05%	0.96	0.12
GO:0030983	mismatched DNA binding	0.22%	0.96	0.18
GO:0003860	3-hydroxyisobutyryl-CoA hydrolase activity	0.02%	0.84	0.18
GO:0004013	adenosylhomocysteinase activity	0.02%	0.85	0.18
GO:0004635	phosphoribosyl-AMP cyclohydrolase activity	0.02%	0.84	0.18
GO:0004636	phosphoribosyl-ATP diphosphatase activity	0.02%	0.85	0.18
GO:0004650	polygalacturonase activity	0.02%	0.85	0.18
GO:0005319	lipid transporter activity	0.58%	0.94	0.19
GO:0098599	palmitoyl hydrolase activity	0.03%	0.84	0.19
GO:0000906	6,7-dimethyl-8-ribityllumazine synthase activity	0.02%	0.83	0.19
GO:0003870	5-aminolevulinate synthase activity	0.02%	0.83	0.19
GO:0003871	5-methyltetrahydropteroyltriglutamate-homocysteine S-methyltransferase activity	0.02%	0.83	0.19
GO:0004788	thiamine diphosphokinase activity	0.02%	0.83	0.19
GO:0004802	transketolase activity	0.03%	0.84	0.19
GO:0008184	glycogen phosphorylase activity	0.02%	0.83	0.19
GO:0030975	thiamine binding	0.02%	0.96	0.2
GO:0008199	ferric iron binding	0.02%	0.97	0.2
GO:0019239	deaminase activity	0.20%	0.83	0.22
GO:0016597	amino acid binding	0.15%	0.97	0.23
GO:0008483	transaminase activity	0.37%	0.8	0.23
GO:0032549	ribonucleoside binding	0.05%	0.96	0.24
GO:0050661	NADP binding	0.30%	0.96	0.25
GO:0005509	calcium ion binding	0.46%	0.97	0.25
GO:0070403	NAD+ binding	0.10%	0.95	0.26
GO:0008097	5S rRNA binding	0.08%	0.96	0.26
GO:0030623	U5 snRNA binding	0.08%	0.96	0.27
GO:0071949	FAD binding	0.23%	0.95	0.27
GO:0008312	7S RNA binding	0.12%	0.96	0.28
GO:0016208	AMP binding	0.15%	0.94	0.28
GO:0003725	double-stranded RNA binding	0.15%	0.96	0.29
GO:0051287	NAD binding	0.46%	0.95	0.29
GO:0010181	FMN binding	0.33%	0.95	0.29
GO:0003747	translation release factor activity	0.10%	0.89	0.3
GO:0050660	flavin adenine dinucleotide binding	0.56%	0.95	0.31
GO:0004152	dihydroorotate dehydrogenase activity	0.02%	0.82	0.31
GO:0004352	glutamate dehydrogenase (NAD+) activity	0.02%	0.82	0.31
GO:0004499	N,N-dimethylaniline monooxygenase activity	0.02%	0.83	0.31
GO:0004754	saccharopine dehydrogenase (NAD+, L-lysine-forming) activity	0.02%	0.83	0.31
GO:0008942	nitrite reductase [NAD(P)H] activity	0.02%	0.83	0.31
GO:0009013	succinate-semialdehyde dehydrogenase [NAD(P)+] activity	0.02%	0.82	0.31
GO:0033743	peptide-methionine (R)-S-oxide reductase activity	0.02%	0.82	0.31
GO:0046577	long-chain-alcohol oxidase activity	0.02%	0.82	0.31
GO:0004605	phosphatidate cytidylyltransferase activity	0.03%	0.82	0.31
GO:0008531	riboflavin kinase activity	0.02%	0.81	0.31
GO:0008484	sulfuric ester hydrolase activity	0.03%	0.83	0.32
GO:0042132	fructose 1,6-bisphosphate 1-phosphatase activity	0.02%	0.81	0.32
GO:0008073	ornithine decarboxylase inhibitor activity	0.02%	0.97	0.33
GO:0070917	inositol phosphoceramide synthase regulator activity	0.02%	0.98	0.33
GO:0003958	NADPH-hemoprotein reductase activity	0.03%	0.82	0.33
GO:0004096	catalase activity	0.03%	0.82	0.34
GO:0030599	pectinesterase activity	0.07%	0.82	0.34
GO:0003684	damaged DNA binding	0.41%	0.96	0.36
GO:0016701	oxidoreductase activity, acting on single donors with incorporation of molecular oxygen	0.08%	0.81	0.36
GO:0004618	phosphoglycerate kinase activity	0.02%	0.81	0.36
GO:0004631	phosphomevalonate kinase activity	0.02%	0.81	0.36
GO:0016780	phosphotransferase activity, for other substituted phosphate groups	0.23%	0.79	0.37
GO:0015109	chromate transmembrane transporter activity	0.04%	0.94	0.37
GO:0001671	ATPase activator activity	0.37%	0.98	0.38
GO:0000155	phosphorelay sensor kinase activity	0.02%	0.78	0.38
GO:0004594	pantothenate kinase activity	0.02%	0.81	0.38
GO:0016303	1-phosphatidylinositol-3-kinase activity	0.02%	0.81	0.38
GO:0008897	holo-[acyl-carrier-protein] synthase activity	0.05%	0.8	0.38
GO:0008478	pyridoxal kinase activity	0.03%	0.81	0.39
GO:0016638	oxidoreductase activity, acting on the CH-NH2 group of donors	0.13%	0.81	0.4
GO:0008409	5'-3' exonuclease activity	0.27%	0.79	0.4
